# A Combined Perceptual, Physico-Chemical, and Imaging Approach to ‘Odour-Distances’ Suggests a Categorizing Function of the *Drosophila* Antennal Lobe

**DOI:** 10.1371/journal.pone.0024300

**Published:** 2011-09-09

**Authors:** Thomas Niewalda, Thomas Völler, Claire Eschbach, Julia Ehmer, Wen-Chuang Chou, Marc Timme, André Fiala, Bertram Gerber

**Affiliations:** 1 Biozentrum, Neurobiologie und Genetik, Universität Würzburg, Würzburg, Germany; 2 Institut für Biologie, Universität Leipzig, Genetik, Leipzig, Germany; 3 Network Dynamics Group, Max Planck Institute for Dynamics and Self-Organization, Göttingen, Germany; 4 Fakultät für Physik, Georg-August-Universität Göttingen, Göttingen, Germany; 5 Bernstein Center for Computational Neuroscience, Göttingen, Germany; 6 Abteilung Molekulare Neurobiologie des Verhaltens, Johann-Friedrich-Blumenbach-Institut für Zoologie und Anthropologie, Georg-August-Universität Göttingen, c/o ENI-G, Göttingen, Germany; 7 Abteilung Genetik von Lernen und Gedächtnis, Leibniz Institut für Neurobiologie (LIN), Magdeburg, Germany; 8 Verhaltensgenetik, Institut für Biologie, Otto von Guericke Universität Magdeburg, Magdeburg, Germany; AgroParisTech, France

## Abstract

How do physico-chemical stimulus features, perception, and physiology relate? Given the multi-layered and parallel architecture of brains, the question specifically is *where* physiological activity patterns correspond to stimulus features and/or perception. Perceived distances between six odour pairs are defined behaviourally from four independent odour recognition tasks. We find that, in register with the physico-chemical distances of these odours, perceived distances for 3-octanol and *n*-amylacetate are consistently smallest in all four tasks, while the other five odour pairs are about equally distinct. Optical imaging in the antennal lobe, using a calcium sensor transgenically expressed in only first-order sensory or only second-order olfactory projection neurons, reveals that 3-octanol and *n*-amylacetate are distinctly represented in sensory neurons, but appear merged in projection neurons. These results may suggest that within-antennal lobe processing funnels sensory signals into behaviourally meaningful categories, in register with the physico-chemical relatedness of the odours.

## Introduction

A flourishing period of research over the past three decades has led to a reasonably detailed picture of how different odours can cause different activity patterns along the olfactory pathway [reviewed in 1]–[5]. In insects, odours are detected by sensory neurons housed within sensillae on the third antennal segment and the maxillary palps. These sensory neurons project to the antennal lobes, the functional equivalent of the olfactory bulb in vertebrates. Each sensory neuron typically expresses one functional *Or* receptor gene, endowing different types of sensory neuron with partially overlapping ligand profiles [6], [7]. Those sensory neurons expressing a common *Or* receptor gene then converge onto one glomerulus within the antennal lobe [8], [9]. For different odours, this entails combinatorially different activity patterns of glomeruli [10]–[12]. Within the antennal lobe, local circuits that comprise interneurons and projection neurons shape the olfactory signal [reviewed in 13]. From the antennal lobe the projection neurons, corresponding to the mitral cells in vertebrates, relay to the lateral horn, a presumed premotor center, as well as to the Kenyon cells of the mushroom body [14]–[17], which may be viewed as a ‘cortical’ structure [18]. Output from the mushroom bodies then projects to presumed premotor areas as well [19]–[21]. Here we ask at which stage of this pathway neuronal activity patterns correspond to perception in the fly [for a pioneering study in the bee: 22].

We define perception in behavioural terms: If two stimuli are perceived differently, these differences should enable the fly to differentially behave towards them. We first provide such an operationally defined, behavioural account of perceived distance between odours. Then, we ask at which stage along the olfactory pathway a fit is found between odour-evoked activity patterns and the salient features of these behavioural measures of perception.

## Results

### A behavioural handle on perceived difference

**Figure 1:**
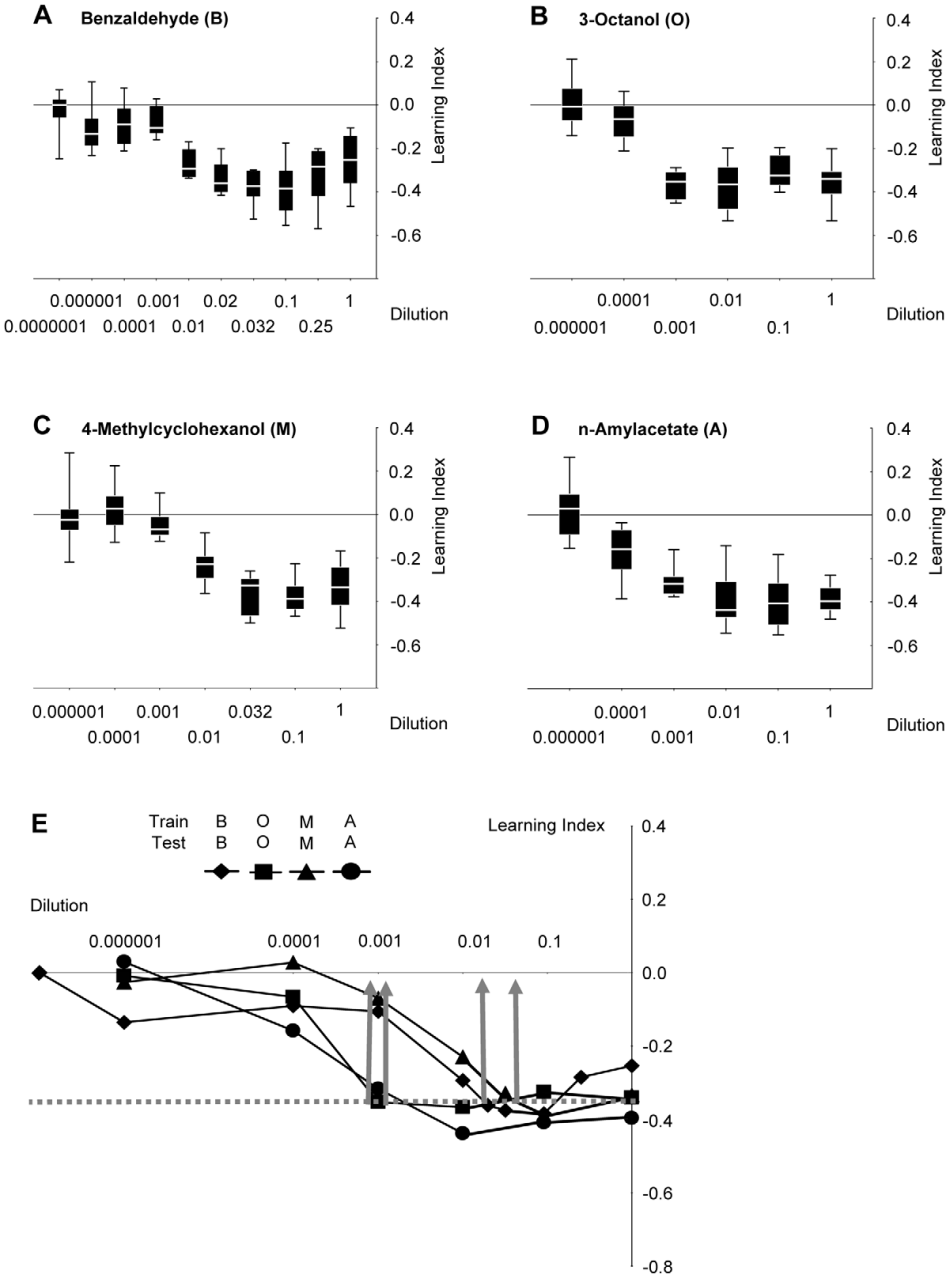
Adjustment of odour intensity for equal learnability. (**A, B, C, D**) Flies are trained with a given odour at the indicated dilution, and then are tested using that same odour at that same dilution. Sample sizes are for B: 12, 12, 12, 12, 12, 12, 12, 8, 8, 8; for O: 11, 12, 12, 12, 8, 8; for M: 12, 8, 12, 12, 12, 8, 8; for A: 12, 12, 12, 12, 8, 8. Data are presented as box plots (middle line: median; box boundaries and whiskers: 25%/75% and 10%/90% quantiles). (**E**) Median data from (**A, B, C, D**) combined. Note that while asymptotic learning scores do not differ between dilutions, the dilutions at which that asymptote is reached differ between odours across almost two orders of magnitude. Dilutions for further experiments are chosen such that learning indices are the same and, for each kind of odour, have just about reached asymptotic levels (stippled grey line and grey arrows) (B: 1∶66; O: 1∶1000; M: 1∶25; A: 1∶1000). For sample sizes, see (**A, B, C, D**).

Our approach was to ask whether flies perceive a test odour *as the same* or *as different* from a previously learned olfactory stimulus. Therefore, dose-effect functions of learnability first needed to be determined, such that odour concentrations could be chosen that support equal learnability for all odours (Fig. 1, 2A). This is important to ensure symmetry of similarity judgements (see Discussion). Also, to keep reasonably clear of task-specific confounds, we used four behavioural tasks (i-iv below) to ‘distill’ the salient, task-independent perceptual relations between odour pairs. We therefore needed to choose relatively few odours, and decided for those that have in the past been used most frequently in the field (benzaldehyde: B; 3-octanol: O; 4-methylcyclohexanol: M; *n*-amylacetate: A).

**Figure 2:**
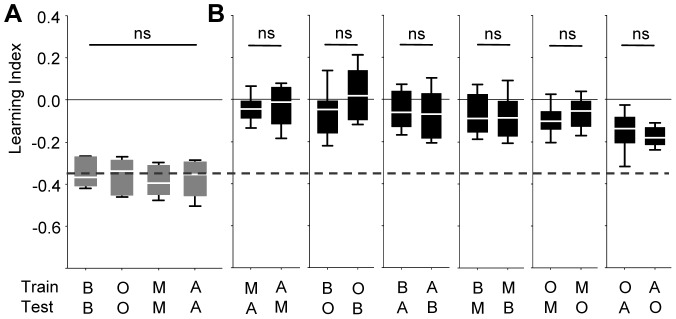
Symmetry of perceived distance. Learning indices dependent on the combination of TRAINing *versus* TESTing odour (benzaldehyde: B, 3-octanol: O, 4-methylcyclohexanol: M, *n*-amylacetate: A). In (**A**), flies were tested with the trained odour, whereas in (**B**) they were tested with a not previously trained odour. Odour-intensities had been chosen for equal learnability (see Fig. 1). The stippled line in (**B**) represents the median of the pooled data from (**A**). Learning indices in (**B**) are in all cases symmetrical, in the sense that scores are equal when e.g. O was trained and A is tested as compared to when A was trained and O is tested. ns: in (**A**) P>0.05 in a Kruskal-Wallis test, in (**B**) P>0.05/6, Mann-Whitney U-tests using a Bonferroni correction. Sample sizes are from left to right: 11, 12, 11, 11, 16, 16, 16,16, 16, 16, 16, 16, 16, 15, 16, 16.

#### Tasks (i) & (ii)

**Figure 3:**
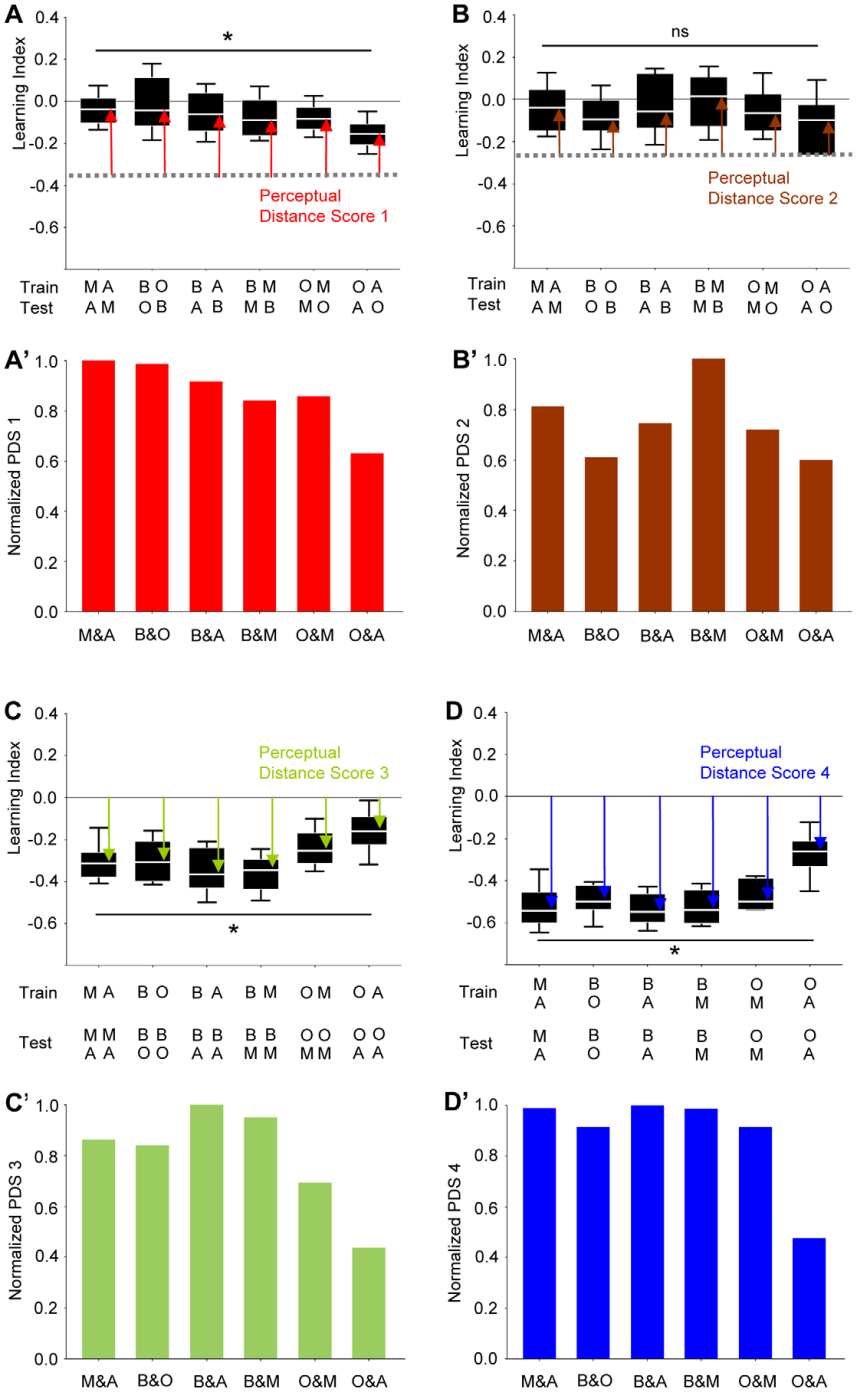
Concordance of perceived distance across four types of recognition experiment. (**A**) Re-presenting the data from Figure 2B, pooled for odour pairs. The stippled grey line represents the learning indices that were found when TRAINing and TESTing odour were identical (from Fig. 2A). To the extent that flies regarded the TESTing odour as different from the TRAINing odour, learning indices should approach zero; thus, the degree to which flies regarded both odours as different can be quantified by the Perceptual Distance Score 1 (red arrows). In (**A′**) these scores were presented normalized to the highest median score thus obtained. Sample sizes are from left to right: 32, 32, 32, 32, 31, 32. (**B**) Same as in (**A**), except that a 180-min break was given between training and test. Sample sizes are from left to right: 24, 24, 24, 24, 24, 24. (**C**) Flies were trained with a given odour, and then were tested for their choice between that trained odour *versus* a novel, not previously trained odour. Thus, if the flies regarded the two TESTing odours as the same, scores should be zero. To the extent that both odours, however, were regarded as different by the flies, learning indices should increase. The level of perceived difference thus can be approximated by the Preceptual Distance Score 3 (green arrows). In (**C′**) these scores are presented normalized to the highest median value thus obtained. Sample sizes are from left to right: 24, 24, 20, 23, 24, 24. (**D**) Flies were trained such that one odour was punished but the other odour was not punished; then, flies were tested for their choice between these two odours. Thus, if the flies could not tell the two odours apart, scores should be zero. To the extent that both odours, however, could be discriminated by the flies, learning indices should increase. The level of perceived difference thus could be approximated by the Preceptual Distance Score 4 (blue arrows). In (**D′**) these scores were presented normalized to the highest median value thus obtained. Sample sizes are from left to right: 15, 11, 12, 11, 11, 12. * and ns refer to P<>0.05 in Kruskal-Wallis tests. Other details as in Fig. 1.

Flies were trained by presenting an odour together with electric shock and then were tested either for their avoidance of that trained odour (Fig. 2A) or for their avoidance of a novel, not previously experienced odour (Fig. 2B) (in this as well as in all following tasks, flies were trained and tested only once). When novel odours were used for testing, learning scores were in all cases symmetrical (Fig. 2B): Scores were equal when e.g. 3-octanol (O) was trained and *n*-amylacetate (A) was tested as compared to the case when A was trained and O was tested (the two right-most plots in Fig. 2B). We therefore pooled the respective subgroups for further analyses. It turned out that in most cases hardly any learned behaviour was observed towards novel odours, reflecting perceived dis-similarity between trained and tested odour. To quantify this perceptual dissimilarity, we determined a ‘Perceptual Distance Score’: If training and testing odours are actually identical (perceived distance is zero), we found learning indices as corresponding to the stippled line in Fig. 3A. We reasoned that to the extent that perception of the test odour deviates from the trained odour (i.e. perceived distance between them increases), the smaller learning indices should be found. Thus, the degree to which learning indices were degraded by presenting a non-trained odour could be used to estimate perceptual distance scores (arrows in Fig. 3A). We noted that for training with O or A allowed the respective other odour to elicit the highest learning scores, both (i) when scores were taken immediately (Fig. 3A, A′) and (ii) when they were taken after an additional retention period of 180 min (Fig. 3B, B′) (see Fig. S1 for the symmetry of the 180-min scores). We interpreted such behaviour as reflecting perceived similarity between these two odours.

#### Task (iii)

We trained flies with joint presentations of one odour with electric shock and then tested the flies for their choice between that trained odour *versus* a novel odour. To the extent that the flies regarded the two odours as different, they should have distributed unequally between them. Thus, in this experiment, perceived distance between the choice-odours should have shown as large learning score (Fig. 3C, C′). We found that perceived distance was smallest between O and A also in this kind of assay (Fig. 3C, C′) (see Fig. S2 for the symmetry of the scores).

#### Task (iv)

We trained flies to discriminate between two odours, such that during training one of the two odours was presented together with electric shock, whereas the other odour was presented without shock. At test we then presented both odours in a choice situation. The more different both odours were regarded by the flies, the easier it should have been to make a difference between them. Thus, perceived distance should have translated into easy discrimination and hence high learning scores (Fig. 3D, D′). We find that again flies regarded O and A as least distant.

**Figure 4:**
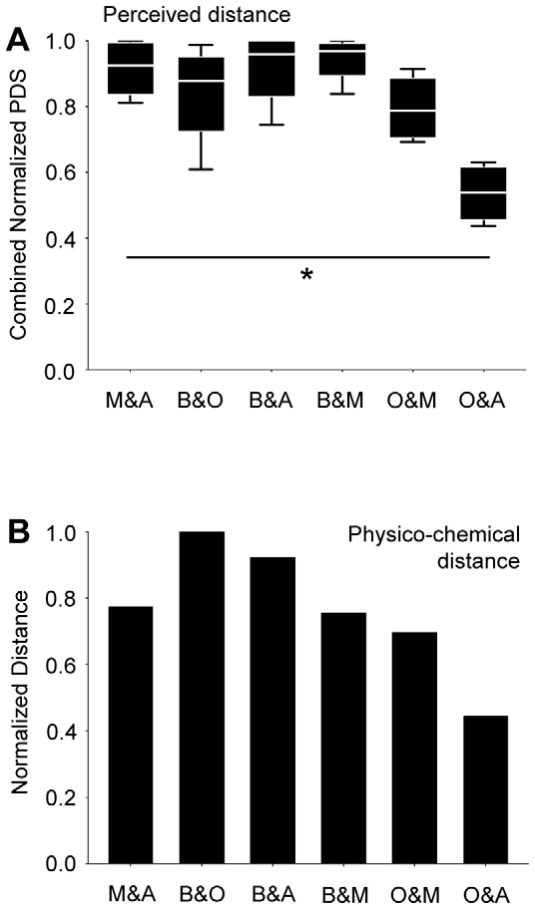
Perceptual and physico-chemical distances. (**A**) The normalized Perceptual Distance Scores (from Fig. 3A′–D′) were combined for each odour pair and presented as box plot. Note the small perceived distance between O and A. * refers to P<0.05 in a Kruskal-Wallis test; N = 4, 4, 4, 4, 4, 4. (**B**) Distances between odour pairs were derived from a physico-chemical description [23]; O and A appeared particularly similar in this kind of analysis, too. Other details as in Fig. 1.

We then combined the normalized perceived distance scores from all four tasks (Fig. 3A′–D′), and derived their median to yield a task-independent perceived distance score for each odour pair (Fig. 4A). This showed that O and A were consistently regarded as the least distant. Because the likelihood for *any one* odour pair having the smallest distance *in all four tasks* is P = 1×1/6×1/6×1/6 = 0.004, we believe that independent of task, O and A reliably have the lowest perceptual distance of our odour set.

When the physico-chemical distances between odour pairs, which consider a large number of molecular properties [23] were calculated, we noted that the smallest distance in these physico-chemical scores was found for O and A, too (Fig. 4B). This prompted us to enquire into the similarity of the patterns of physiological activity evoked by these odours.

### Physiology

The DNA-encoded fluorescence calcium sensor cameleon 2.1 [10], [24], [25] was expressed either in large populations of first- or in second-order olfactory neurons, i.e. either in sensory neurons or in projection neurons. Odour-evoked increases in calcium levels in these respective populations of cells were measured at the antennal lobes, the site where the sensory neurons relay onto the projection neurons. To avoid potential intensity artefacts we used the same odorant dilutions as for the behavioural experiments. Each individual fly was presented with all four odours. On the one hand, this enabled us to determine, for each animal and odour pair, the distances between the evoked activity patters (see below). On the other hand, the requirement to probe each fly with all odours limited the total number of odours that could reasonably be included in such an analysis.

**Figure 5:**
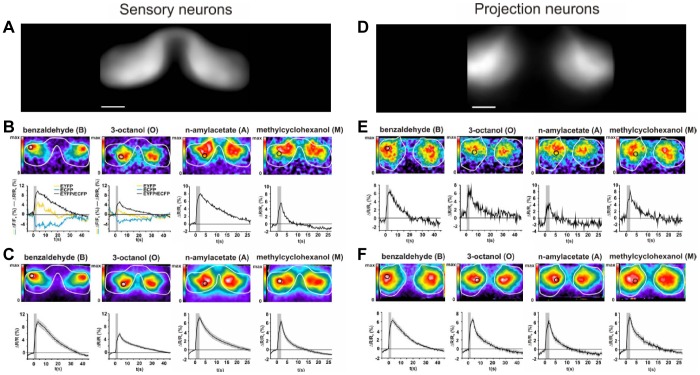
High signal quality and low inter-individual variability in physiology. (**A**) To illustrate the shape of the antennal lobe as apparent in measurements of the sensory neurons, EYFP emission averaged across 8 individual flies is presented. Scale bar 25 µm. (**B**) Calcium activity recorded in the antennal lobes (white circumference-line) in sensory neurons of an individual fly after a single stimulation with the indicated odours, displayed in false-colour (top). For a defined region of interest (black circle), the time course of the measurements is displayed (bottom) as the EYFP/ECFP ratio (black). For benzaldehyde and 3-octanol as examples, also the EYFP (yellow) and ECFP (cyan) signals are plotted. The grey bar indicates the duration of the odour stimulus. (**C**) Calcium activity in olfactory sensory neurons averaged across 3–5 stimulations for each odour and in 8 individual animals displayed in false-colour (top). For the region of interest (black circle), the time course of calcium activity is displayed for the ration EYFP/ECFP (bottom). The grey bar indicates the duration of the odour stimulus. Data represent mean ± SEM. (**D, E, F**) Same as **A**, **B**, **C**, but for antennal lobe-measurements of projection neuron activity.

**Figure 6:**
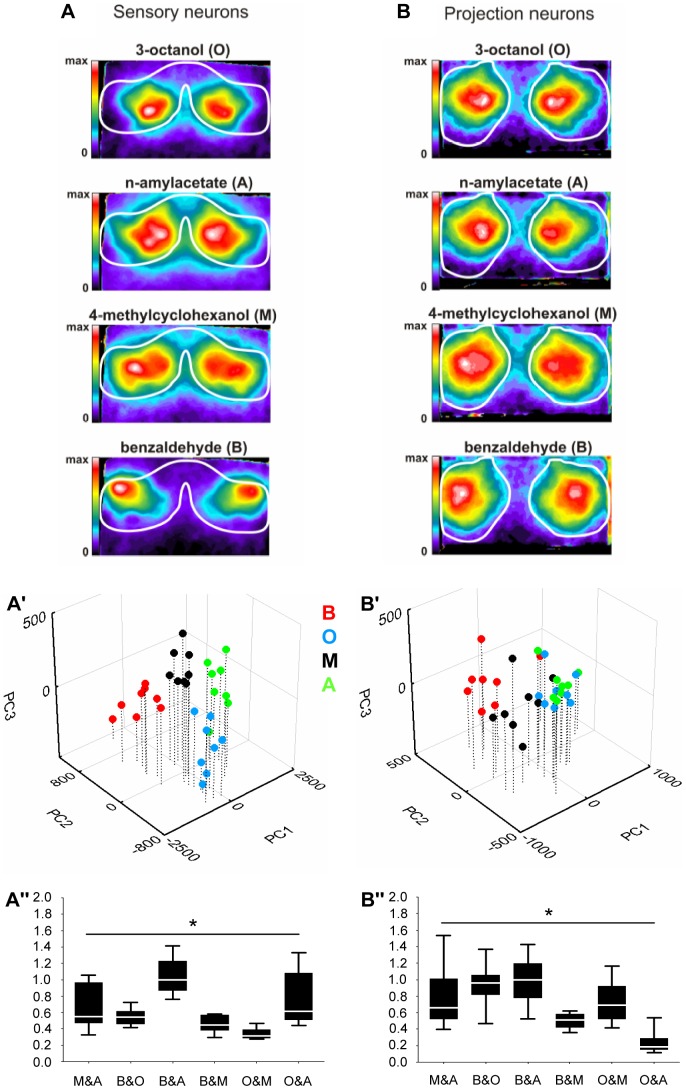
Quantitative analysis of activity patterns in first- and second-order olfactory neurons. False-colour coded calcium activity patterns in the antennal lobes (**A, B**), the respective PCAs (**A′, B′**), and Euclidian PCA distances (**A″, B″**) evoked by four different odorants in sensory neurons (**A- A″**) or projection neurons (**B- B″**). (**A, B**) Images present averages of eight individual flies, and 3–5 stimulations with the respective odour. Data are normalized to the maximum signal of the averaged image. The white lines indicate the outline of the antennal lobes as labelled by the respective Gal4-line (*Dmel/Orco*-Gal4, formerly known as *Or83b*-Gal4,or *GH146*-Gal4, respectively). Note that in the sensory neurons, the activity pattern evoked by O is nested within the one evoked by A; however, in the projection neurons O and A evoke the same pattern of activity. Please note that **A** and **B** re-present the data from Fig. 5 C and F, respectively. (**A′, B′**) Pixel-wise principal component analyses across odour-evoked calcium activity within the antennal lobes as measured from sensory neurons (**A′**) or projection neurons (**B′**). Different colours indicate different odorants as indicated. Each coloured circle indicates a measurement of an individual animal. Note that in projection neurons, but not in sensory neurons, the activity patterns evoked by O and A coincided. (**A″, B″**) Euclidian distances on the basis of the first three principal components for each pair of odours were determined for each fly; these distances were combined across flies, and displayed normalized to the highest median distance thus obtained. O and A did not appear particularly similar in sensory neurons (**A″**), but turned out as the least distant odour pair in projection neurons (**B″**). * refers to P<0.05 in Kruskal-Wallis tests probing for differences across all odour pairs; N = 8 in all cases. Other details as in Fig. 1.

Regarding olfactory sensory neurons, Figure 5 shows that calcium signals in the antennal lobe were odour-specific, spatially restricted, bilaterally symmetric, and showed remarkably high signal-to-noise ratio. Glomerular structures, however, cannot be reliably resolved with the employed technique, preventing the identification of the activated glomeruli. However, the odour-evoked patterns of activity were stimulus-specific and consistent across individuals, allowing us to compare the activity patterns, averaged across individual flies, between the four odours. Obviously, the four odours evoked distinct activity patterns at the input stage to the antennal lobe (Fig. 6A), with the activation by O nested within the pattern evoked by A. In order to subject these activity patterns to quantitative analysis, we performed a pixel-wise principal component analysis (PCA), graphically represented by the first three principal components, covering more than 90% of the variability in the dataset (Fig. S3). In such a PCA, data from the eight experimental flies clustered separately for each of the four odorants (Fig. 6A′). Notably, this PCA did not uncover a particularly low distance between O and A on the sensory neuron level (Fig. 6A″).

What about the projection neurons? Odour-evoked activity patterns for O, M, and B were more widely distributed across the antennal lobe when compared to the sensory neurons (e.g. Fig. 5B *versus* Fig. 5E) and appeared less consistent between individual flies (see below). Activity patterns, however, still were sufficiently local and conserved across individual flies to allow averaging across animals and comparing these averaged activity patterns between odours (Fig. 6B). A PCA confirmed that data of individual odours were distributed relatively more widely than is the case for the sensory neurons, reflecting the above-mentioned higher inter-individual variability (Fig. 6B′) and presumably also the more widely distributed arborisations of projection neurons in the antennal lobe. Importantly, in this projection-neuron based PCA approach, the data for O and A formed one merged cluster (Fig. 6B″).

**Figure 7:**
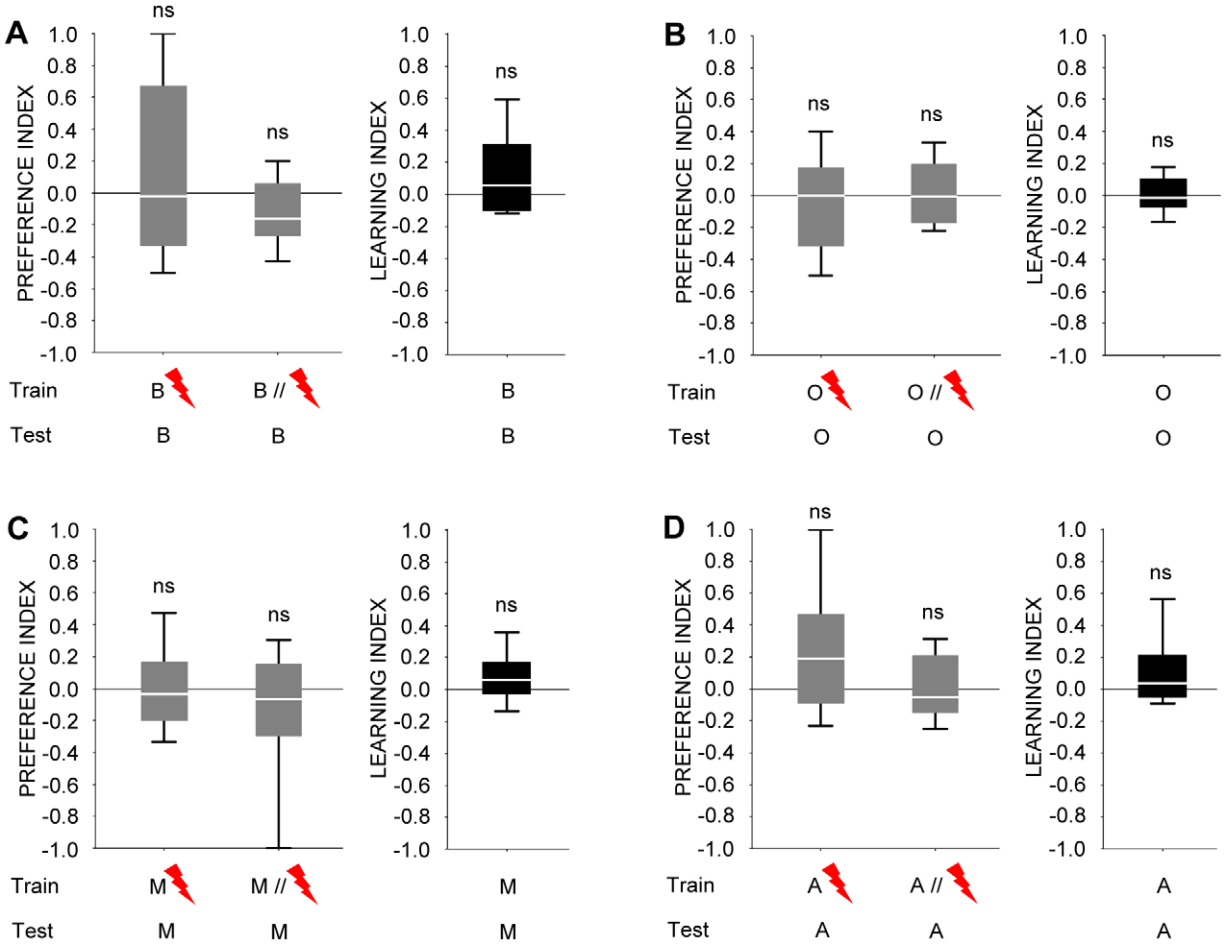
A *Dmel*/*Orco* loss-of-function mutant (formerly known as *Or83b^2^*) is anosmic for all odours used. (**A**), (**B**), (**C**), and (**D**) show preference indices (grey fill) for respectively benzaldehyde, 3-octanol, 4-methylcyclohexanol, and *n*-amylacetate after odour-shock training, and the corresponding learning indices (black fill). Neither preference indices nor learning indices are different from zero in the *Dmel*/*Orco* mutant, suggesting an absolute requirement of *Dmel*/*Orco* for processing of these odours. Thus, a lack of correspondence between perception and sensory-neuron physiology cannot be attributed to processing outside of the *Dmel*/*Orco*-Gal4 expression pattern.

Thus, the low perceptual distance for O and A (Fig. 4A) did not apparently conform to sensory-neuron distances (Fig.′6A″) (this lack of match was not due to processing outside the sensory neuron driver (*Dmel/Orco*-Gal4, the driver formerly known as *Or83b*-Gal4, because *Dmel/Orco* loss-of-function mutants were anosmic for all odours used: Fig. 7). However, in the projection neurons a low distance between O and A was revealed (Fig.′6B″). Therefore, the processing step from first- to second-order olfactory neurons apparently corresponds to a categorization step, making the activity patterns for O and A more similar. In our dataset, this came about by a sharpening of the activity pattern evoked by A such that, while at the level of the sensory neurons the signal evoked by O was nested within the one evoked by A, both odours activated almost fully overlapping areas of the antennal lobe when the projection neurons were considered (Fig. 6).

## Discussion

The relationship between olfactory perception and physiology has been elegantly studied in the honeybee [22]: One out of 16 odours was trained by presenting it together with a sugar reward, and, for any individual bee, testing for conditioned proboscis extension was carried out with a random draw of four from these 16 odours to generate a 16-dimensional behavioural odour space. Euclidian distances between odour pairs could thus be used for a correlation analysis with similarities of physiological activity patterns in the antennal lobe as had been measured earlier using bath-applied calcium dyes [26]. In agreement with what we report here, behavioural and physiological distances between odour pairs matched fairly well. However, using bath-applied dyes does not allow one to assign cellular identity of the measured cells with reasonable certainty. Also, behavioural scores were in a number of cases asymmetrical: Response levels to aldehydes were generally high after training to odours of other functional classes (primary and secondary alcohols, ketones), whereas after training with aldehydes response levels to odours from these other classes were low. Such asymmetries can result from not adjusting odour intensities for equal learnability (and/or from the repeated testing of individual bees). For example, suppose for task (i) an odour X would have high learnability, whereas odour Y would be less learnable at the respective dilution used. One may then find strong conditioned avoidance of Y after training with X because the memory for X is strong and because X and Y are regarded as similar to a particular extent. However, after training with Y, conditioned avoidance of X may be low simply because the memory for Y is weak and although X and Y actually are regarded as similar. This would entail an apparent asymmetry of similarity judgments, violating a fundamental property of metrices in a mathematical sense (the distance between X and Y cannot be different from the distance between Y and X).

Our findings may at first sight appear inconsistent with the report of Kreher et al. [7]. The authors measured odour-induced electrophysiological activity in adult *Drosophila* olfactory sensory neurons which express, rather than their cognate *Or* gene, only one of the 21 larval-expressed *Or* genes. This was done for all these 21 larval *Or* genes and a panel of 26 odours to obtain a physiological odour space. Behaviourally, the authors assayed larval *Drosophila* in a masking experiment: One odour was presented as a point source within a background mask of another odour present throughout the experimental arena. If a larva responds to the point source despite the mask, it must have the ability to tell apart the point source from the mask. Regarding odour quality processing, the argument requires that no behavioural responses to the point source are seen if the same odour is used as both point source and mask. This was shown to be the case for four out of the six odours thus assayed. Notably, results were in some cases asymmetrical (e.g. ethyl butyrate and 2,3-butanedione; see discussion above). Still, perceptual similarity thus measured correlates with the distances between odours in the physiological odour space. This is not a contradiction to our findings, however, because focussing on the sensory neurons may not reveal potentially better matches between physiology and perception in the projection neurons. Also, different sites along the olfactory pathway may be important for different kinds of behavioural similarity judgements: Masking may come about on the level of sensory neurons and thus the physiology of these very neurons may underlie masking-based measures of perception, while more central processing stages may be involved in recognition-type measures of perceived similarity, as in our case.

We note that the distances of odour pairs in perception (Fig. 4A), in terms of physico-chemical distance (Fig. 4B), and projection-neuron physiology (Fig. 6B″) all suggest O and A to be relatively similar. This may imply that the actual physico-chemical parameters of odours are not as such given in sensory neurons, but need to be derived as processing progresses. In the case of O and A, this apparently entails a classification of sensory inputs according to their overall physico-chemical similarity. Perception and ensuing behaviour seem to be based on these processed, second-order categories. Admittedly, the correspondences between perceptual distance, projection-neuron physiological distance, and physico-chemical distance are coarse (see for example the odour pair B and M), within this as well as earlier [7], [22] studies. This may be due to differences in genotype between behavioural and physiological measurements, imperfections and/or incompleteness of physiological measurements, the kind and number of odours sampled, and/or due to specific demands imposed by the respective behavioural assays. Also, processing stages downstream of sensory and projection neurons, such as the mushroom bodies, and/or temporal aspects of physiological activity likely contribute to shape perception. These caveats in mind, finding even a coarse match between perception, physiology at any one processing step, and physico-chemical odour features is actually surprising. The employed widefield microscopy to determine calcium activity patterns in *both* antennal lobes makes it difficult to identify the activated glomeruli because calcium signals are detected from different depths of the preparation. Therefore, we intentionally refrain from referring calcium activity patterns to identified glomeruli. Rather, we apply a more unbiased method and describe the similarity between odour-evoked calcium activity patterns on the basis of pixels. In the future, it will be of interest to use high-resolution microscopy (e.g. 2-photon-imaging) to determine in detail the anatomical substrates as well as the underlying circuit architecture which causes a catergorization of odour stimuli.

Thus, based on our results we suggest that within-antennal lobe processing may organize odour-evoked activity according to the physico-chemical properties of the odours, and that this process may be a basis for the flies' behavioural similarity judgements. Regarding these judgements it seems important to note that along the olfactory sensory-motor loop olfactory signals, gradual in nature, eventually have to be dichotomized by the flies in order to ‘decide’ whether to run away from a given odour- or not. The first steps in this process to funnel olfactory representations into behavioural categories, we suggest, may already be taken at the level of the antennal lobe, according to the physico-chemical properties of the odours. Given that so far the antennal lobe network has mostly been implicated in maintaining or even enhancing distinctiveness between odours [discussion in 13], such a categorization process would provide a novel aspect of antennal lobe function.

## Materials and Methods

### Behaviour

Wild-type Canton-S flies were kept in mass culture at 25°C, 60–70% humidity and a 14/10 hour light/dark cycle. For the data displayed in Fig. 7, an *Dmel/Orco* loss-of-function mutant [27] (the mutant formerly known as *Or83b^2^*) was used (Bloomington stock center, #23130). Flies were collected one to five days after pupal hatching and kept over-night at 18°C.

Training was performed in dim red light, testing in darkness. As stimuli we used benzaldehyde, 3-octanol, 4-methylcyclohexanol, or *n*-amylacetate (B, O, M, A) (CAS: 100-52-7, 589-98-0, 589-91-3, 628-63-7; all from Fluka, Steinheim, Germany, except A, which is from Merck, Darmstadt, Germany), or ambient air (Θ). This odour choice was based on the *Drosophila* learning literature since the 70 s; we thus probably sampled a subset of relatively easily discriminable odour pairs. A vacuum pump ensured removal of odour-saturated air from the training apparatus. Odorants (130 µl) were applied in Teflon cups of 7-mm diameter either in pure condition or diluted in paraffin oil (B: 1∶66; O: 1∶1000; M: 1∶25; A: 1∶1000, unless mentioned otherwise) (paraffin oil from Merck, Darmstadt, Germany). At t = 0 min, groups of about 100 flies were loaded to the training tubes of the experimental apparatus which allowed applying electric shock via an electrifiable grid covering the tube. At t = 2 min, the first stimulus (either B, O, M, A, orΘ) was presented for 60 s without punishment. At t = 4 min, the second stimulus (any of the remaining four) was presented for 60 s; 15 s after stimulus onset, an electric shock was applied (90 volts, 12 pulses á 1.2 s within 60 s, onset-onset interval 5 s). At t = 9∶00 min, flies were transferred back to their food vials for 13 min until the next of the in total three such training cycles starts. Across independent measurements, the sequence of events was either as indicated during all three training cycles, or was reversed such that the first stimulus presented was punished.

After training, the regular 13 min break was given (unless mentioned otherwise). After an accommodation period of 4 min, animals were transferred to an appr. 1.5 cm^3^ choice chamber of a T-maze, from where they could escape towards either of two of the five above-mentioned stimuli. After 2 min, the arms of the maze were closed, the number of animals within each arm (denoted #) counted, and the relative preference between the choice options determined as documented in Fig.s S5, S6, S7, S8, S9. A preference index (PI) was calculated as:
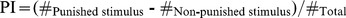
(1)


A second set of flies was trained reciprocally: If e.g. in Experiment (iv) (Fig. 3D), one set of flies was punished when receiving M but not when receiving A, the second set of flies was trained by presenting A with but M without punishment. The same reciprocity was followed in all tasks. PIs of these two reciprocally trained sets of flies were then averaged to obtain a learning index (LI). Thus, positive LIs indicate conditioned approach, negative LIs conditioned avoidance. Data are presented as box plots with the middle line showing the median and box boundaries and whiskers the 25%/75% and 10%/90% quantiles, respectively, and were analyzed with non-parametric statistics (Statistica, Statsoft, Hamburg, Germany), using a Bonferroni correction as applicable. Flies were trained and tested only once.

After adjusting odour dilutions for equal learnability (Fig. 1; Fig. 2A), four tasks were performed:

- In a 4×4×2 experimental design, flies were trained with any one of the four odours *versus* Θ. Then, they were tested either for their avoidance of the trained odour, or of any one of the remaining three non-trained odours, *versus* Θ. This was done either after the regular 13-min break (i), or after an additional 180-min waiting period (ii).
- Flies were trained as in (i), but were tested in a two-odour choice situation for their relative preference between the punished *versus* any of the three non-punished odours.
- Flies were trained differentially between two odours and were then tested for their relative preference between them in a two-odour choice situation.

### Physico-chemical distances

We used the odour metric as presented by [23]. Odour structures were obtained from PubChem (http://pubchem.ncbi.nlm.nih.gov/) and input to the Dragon software (http://www.talete.mi.it/products/dragon_description.htm). In the used version 5.4, this metric represented each odorant as vector of 1664 molecular descriptor values and yielded, for the respective odour pairs, the following values: M-A: 28.6755; B-O: 37.0393; B-A: 34.1564; B-M: 27.9832; O-M: 25.8083; O-A: 16.5091. In Fig. 4B, these scores are presented normalized to the highest value thus obtained. We note that when using a second, independent metric [28], [29], the pattern of results was the same (not shown; pers. comm. Michael Schmuker, Freie Universität Berlin).

### Physiology: Optical calcium imaging

Cameleon 2.1 [25] was expressed using either *Dmel/Orco*-Gal4 (formerly known as *Or83b*-Gal4) [27], or *GH146*-Gal4 [30]. All animals were homozygous for both the UAS:*cameleon* insertion ([31]: strain 82) and the respective Gal4 insertion.

5–7 day-old female flies were briefly cooled on ice for immobilization and restrained by inserting them into a truncated pipette tip with the head sticking out. The fly was glued with its head under a transparency foil and then fixed on a plastic cover slip using dental glue (Protemp II, 3M ESPE, Seefeld, Germany). The third antennal segments and maxillary palps remained dry and untouched. A window was cut into the head capsule and the hole covered by a drop of Ringer's solution [32]. The preparation was placed under an upright widefield fluorescence microscope (Zeiss Axioscope 2 FS) equipped with a 40× water immersion objective (Zeiss Achroplan) (Zeiss, Göttingen, Germany) and a cooled CCD camera (CoolSnap HQ, Photometrics, Pleasanton, CA). Excitation light of 436 nm was provided by a xenon lamp and a grid monochromator (Visitron Systems, Puchheim, Germany). Fluorescence emission was guided through a 455 nm DCLP pass filter (Chroma Technologies, Rockingham, VT, USA); the wavelengths of EYFP and ECFP emission (530 nm and 480 nm, respectively) were separated using a beam splitter (Optical Insights, Santa Fe, NM, USA) equipped with a cameleon filter set (Chroma Technologies, Rockingham, VT, USA). The two half-images of EYFP and ECFP emissions were simultaneously recorded by the two halves of the CCD chip (1392×1040 pixels) at a binning of 4×4, resulting in one stored image of 174×260 pixels per time frame and wavelength. After binning, each stored pixel was a 14-bit real number reflecting the image intensity of the respective wavelength. Images were acquired at a frame rate of 5 Hz (200 ms) with an exposure time of 100 ms per frame, controlled by MetaFluor software (Visitron Systems, Puchheim, Germany). Each EYFP image at time point t was labelled S^Y^(t), and each ECFP image S^C^(t), respectively.

Odour delivery was achieved using a custom-built olfactometer. A constant air stream generated by a vacuum pump was directed via a glass pipette to the fly's antennae and maxillary palps. The airstream was shunted to vials that are either blank, contained paraffin oil as solvent-control or one of the four odorants diluted in paraffin oil as for the above behavioural experiments. All flies received cycles of six stimulations each, in the order blank air, solvent, O, A, B, and M. Specifically, 2-s stimuli are applied 3 s after the onset of the experiment, followed by a 60 s break after which another stimulus was applied until the set of stimulations was complete. This cycle was repeated 3–5 times for each fly.

### Quantitative data analysis

Image alignment was performed using a modified version of the ImageJ plugin TurboReg [33] that allowed for the alignment of images without changing the value of any pixel. First, images were cropped by 5 pixels in one direction to remove a black edge produced by the beam splitter device, resulting in 169×260 pixels per image. Data analysis then was performed using a custom-written Java script implemented in ImageJ. Aligned EYFP and ECFP images were used to generate EYFP/ECFP ratio images S(t) = S^Y^(t)/S^C^(t); all subsequent image analysis was based on this ratio signal. For calculating odour-evoked calcium signals, five frames preceding odour onset (frame 8–12; odour onset at frame 16) were averaged (prestimulus), and five frames beginning 400 ms after odour onset (frames 18–22) were averaged (stimulus). The averaged prestimulus image then was subtracted from the averaged stimulus image to obtain a calcium signal image. To reduce noise, images were filtered by replacing each pixel intensity by the average of the surrounding 8×8-pixel area. To reduce noise, the calcium signal images obtained by the 3–5 stimulations per odour were averaged for each fly measured.

Time courses of calcium signals averaged over distinct regions of interest (defined in the figures) were calculated based on the original images S^Y^(t) and S^C^(t) using the MetaMorph software (Visitron Systems, Puchheim, Germany). For time-resolved estimates of calcium activity (e.g. bottom of Fig. 5B, E), fluorescent emission of EYFP and ECFP averaged over a distinct region outside the labelled structure (the ‘background’ outside the white circumfence line of e.g. top of Fig. 5B, marked in the respective figures) was at each time point subtracted from the value within the chosen region of interest (F(t)-value: either F^Y^(t) or F^C^(t)) (e.g. black circle in Fig. 5B, top). For calculating changes in fluorescence (ΔF(t)), the F(t) value at odour onset (F_0_) was subtracted from the F(t) value at the respective time point t; ΔF(t) was then divided by F_0_ (ΔF(t)/F_0_). To exploit the sensors' nature of increasing EYFP fluorescence and decreasing ECFP fluorescence upon increased calcium levels, which largely eliminates movement artefacts, the ratio of F(t)-values for EYFP and ECFP was calculated (EYFP/ECFP) (R(t)-value: R(t) = F^Y^(t)/F^C^(t)); thus, the normalized change in this ratio (ΔR(t)/R_0_) represented calcium activity. Maximum calcium activity was typically found in a time window 3 s after odour onset (e.g. bottom of Fig. 5B); thus, the false-colour coded images (e.g. top of Fig. 5B) represent calcium activity (ΔR(t)/R_0_) for each pixel at this time point.

For analyzing odour-evoked calcium activity patterns, the regions of interest (ROI) covering one antennal lobe in calcium images S^Y^(t) and S^C^(t) were first defined using thresholding (Fig. S4). Pixel intensities of background EYFP images were averaged and are normalized between 0 and 1 and were chosen as ROI pixels when intensities are greater than 0.40 or 0.65 for sensory neurons or projection neurons, respectively. The choice of threshold values depends on the contour of the ROI, reflecting the anatomical position of the investigated groups of neurons. Only the calcium signals within the ROI were used for further analysis.

We used Principle Component Analysis (PCA) to reduce the high-dimensional data to three dimensions that accounted for most of the variance. The principle components (PCs) were indexed according to their contribution to the total variance. Here, the calcium signals in the ROI (7575 data points for sensory neurons and 5890 data points for the projection neurons, respectively) were reduced to the three dominant principle components that turned out to keep >90% of the variability of the signals (see Fig. S3). Euclidian distances were computed for each pair of odours based on the first three PCs, combined across flies and displayed as box plots in Fig. 6A″, B″ normalized to the highest median distance thus obtained.

## Supporting Information

Figure S1 **Symmetry of perceived distance measures.** (**A**) Confirming that also after an additional retention period of 180 min learning indices are equal for the chosen dilutions of odour. Sample sizes are from left to right 8, 8, 8, 8. (**B**) Data from Fig. 3B separated by odour; note that learning indices in all cases are symmetrical, in the sense that response levels e.g. to A after training with O are as high as response levels to O after training to A. The stippled line in (**B**) represents the median of the pooled data from (**A**) and corresponds to the one in Fig. 3B. Sample sizes are from left to right 12, 12, 12, 12, 12, 12, 12, 12, 12, 12, 12, 12. Other details, and abbreviations of odour identity, as in Fig. 1.
(TIF)

Figure S2 **Symmetry of perceived distance measures.** Data from Fig. 3C, separated by odour. Note that learning indices in all cases are symmetrical, in the sense that learning scores are the same when choice between O and A is assayed after training to O, as they are after training to A. Sample sizes are from left to right 12, 12, 12, 12, 10, 10, 11, 12, 12, 12, 12, 12. Other details, and abbreviations of odour identity, as in Fig. 1.
(TIF)

Figure S3 **Validation of the three-PC based Euclidian distance measures.** Euclidian distances of odour-evoked activity (**A**: sensory neurons, **B**: projection neurons) are computed for each pair of odours based on increasing numbers of principle components (x-axis: #PCs). The differently colored lines indicate data from individual animals. Note that for both populations of neurons the Euclidian distances remain constant or only slightly increase when using more than three principle components, demonstrating that the relative similarity between calcium activity patterns is effectively covered by the first three principle components. In other words, additional principle components do not add significant information.
(TIF)

Figure S4 **Definition of the Region of Interest (ROI) for the pixel-based PCA.** (**A**) To define the Region of Interest (ROI) for the PCA of the sensory neurons innervating the antennal lobes across measurements, EYFP emission across 8 individual flies is averaged. (**B**) The region of interest used for PCA of sensory neuron activity in the antennal lobe (red circumference-line), defined by using a threshold of 0.45 of the maximum intensity value. (**C**) As in (**A**), but for the projection neurons. (**D**) As in (**B**), but for the projection neurons, except that (**C, D**) used a threshold of 0.60 of the maximum intensity value.
(TIF)

Figure S5
**Preference scores underlying the associative performance indices shown in** **Figure 1A–D** **.** The behaviour of the reciprocally trained groups of flies as underlying the associative learning indices (LIs) of Fig. 1A–D is documented by preferences (PREF) scores. On the basis of the the number of flies in the respective arm of the maze (#) these scores are calculated as:
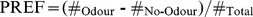

(TIF)

Figure S6
**Preference scores underlying the associative performance indices shown in** **Figure 2A–B** **.** The behaviour of the reciprocally trained groups of flies as underlying the associative learning indices (LIs) of Fig. 2A–B is documented by preferences (PREF) scores. On the basis of the the number of flies in the respective arm of the maze (#) these scores are calculated as:
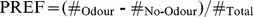

(TIF)

Figure S7
**Preference scores underlying the associative performance indices shown in** **Figure 3D** **.** The behaviour of the reciprocally trained groups of flies as underlying the associative learning indices (LIs) of Fig. 3D is documented by preferences (PREF) scores. On the basis of the the number of flies in the respective arm of the maze (#) these scores are calculated as:
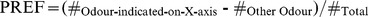

(TIF)

Figure S8
**Preference scores underlying the associative performance indices shown in Figure S1A–B.** The behaviour of the reciprocally trained groups of flies as underlying the associative learning indices (LIs) of Fig. S1A–B is documented by preferences (PREF) scores. On the basis of the the number of flies in the respective arm of the maze (#) these scores are calculated as:
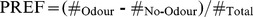

(TIF)

Figure S9
**Preference scores underlying the associative performance indices shown in Figure S2.** The behaviour of the reciprocally trained groups of flies as underlying the associative learning indices (LIs) of Fig. S2 is documented by preferences (PREF) scores. On the basis of the the number of flies in the respective arm of the maze (#) these scores are calculated as:
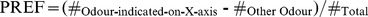

(TIF)
