# Supplementary figures and images for: A Combined Perceptual, Physico-Chemical, and Imaging Approach to ‘Odour-Distances’ Suggests a Categorizing Function of the Drosophila Antennal Lobe
Source: PLoS One. 2011 Sep 9;6(9):e24300. doi: 10.1371/journal.pone.0024300 (PMC3170316; doi:10.1371/journal.pone.0024300)

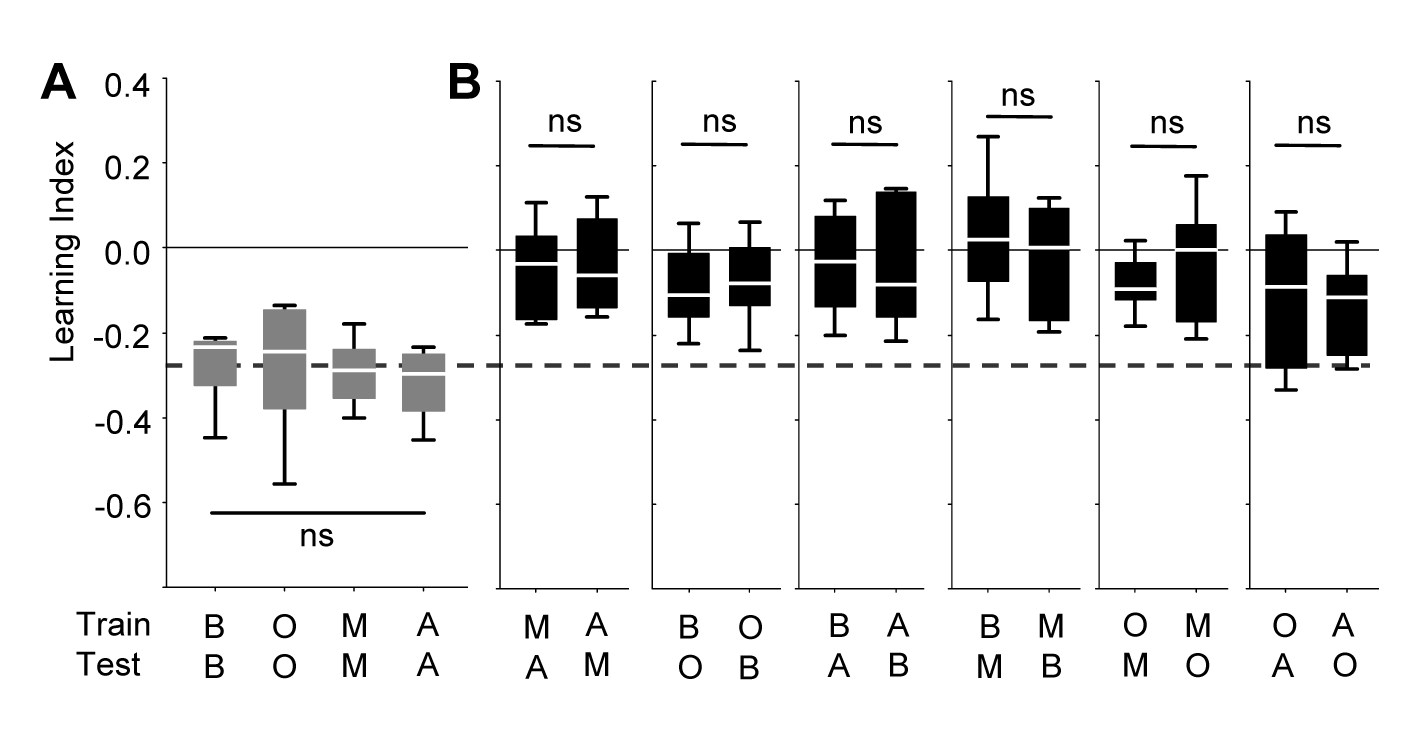

Supplement: Figure S1 — Symmetry of perceived distance measures. (A) Confirming that also after an additional retention period of 180 min learning indices are equal for the chosen dilutions of odour. Sample sizes are from left to right 8, 8, 8, 8. (B) Data from Fig. 3B separated by odour; note that learning indices in all cases are symmetrical, in the sense that response levels e.g. to A after training with O are as high as response levels to O after training to A. The stippled line in (B) represents the median of the pooled data from (A) and corresponds to the one in Fig. 3B. Sample sizes are from left to right 12, 12, 12, 12, 12, 12, 12, 12, 12, 12, 12, 12. Other details, and abbreviations of odour identity, as in Fig. 1. (TIF) [file pone.0024300.s001.tif]

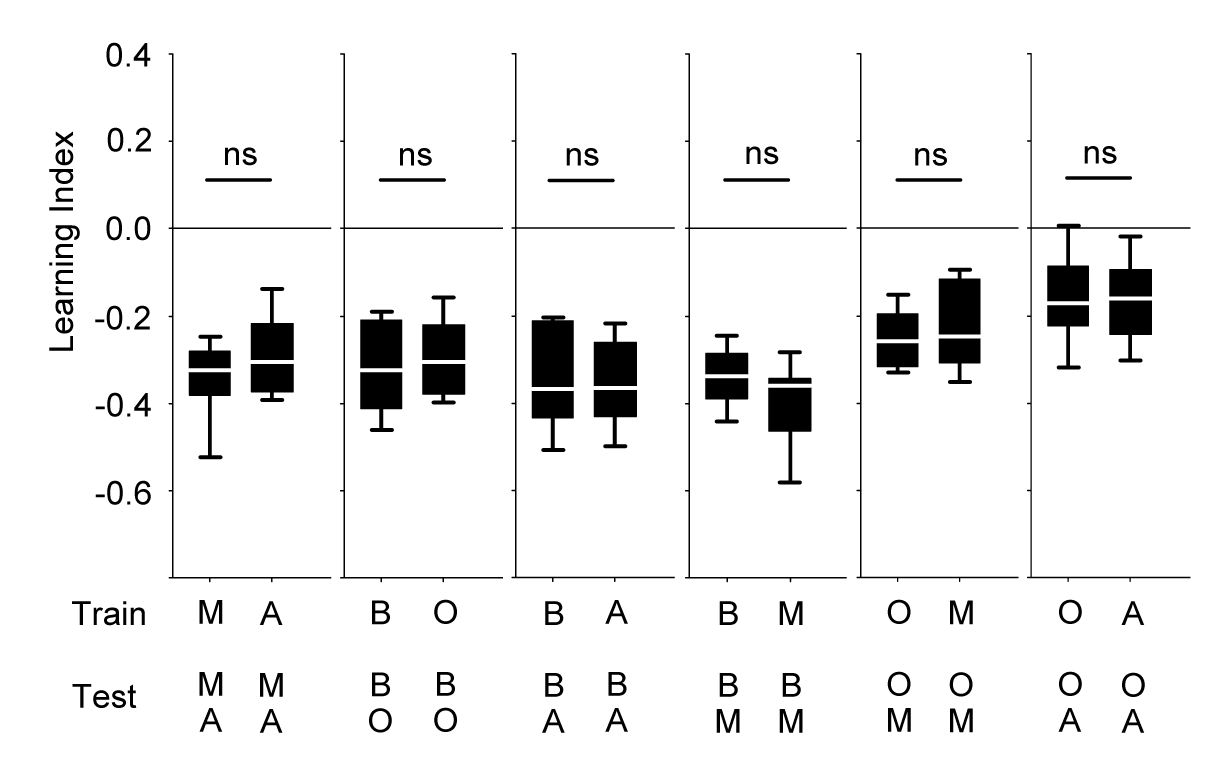

Supplement: Figure S2 — Symmetry of perceived distance measures. Data from Fig. 3C, separated by odour. Note that learning indices in all cases are symmetrical, in the sense that learning scores are the same when choice between O and A is assayed after training to O, as they are after training to A. Sample sizes are from left to right 12, 12, 12, 12, 10, 10, 11, 12, 12, 12, 12, 12. Other details, and abbreviations of odour identity, as in Fig. 1. (TIF) [file pone.0024300.s002.tif]

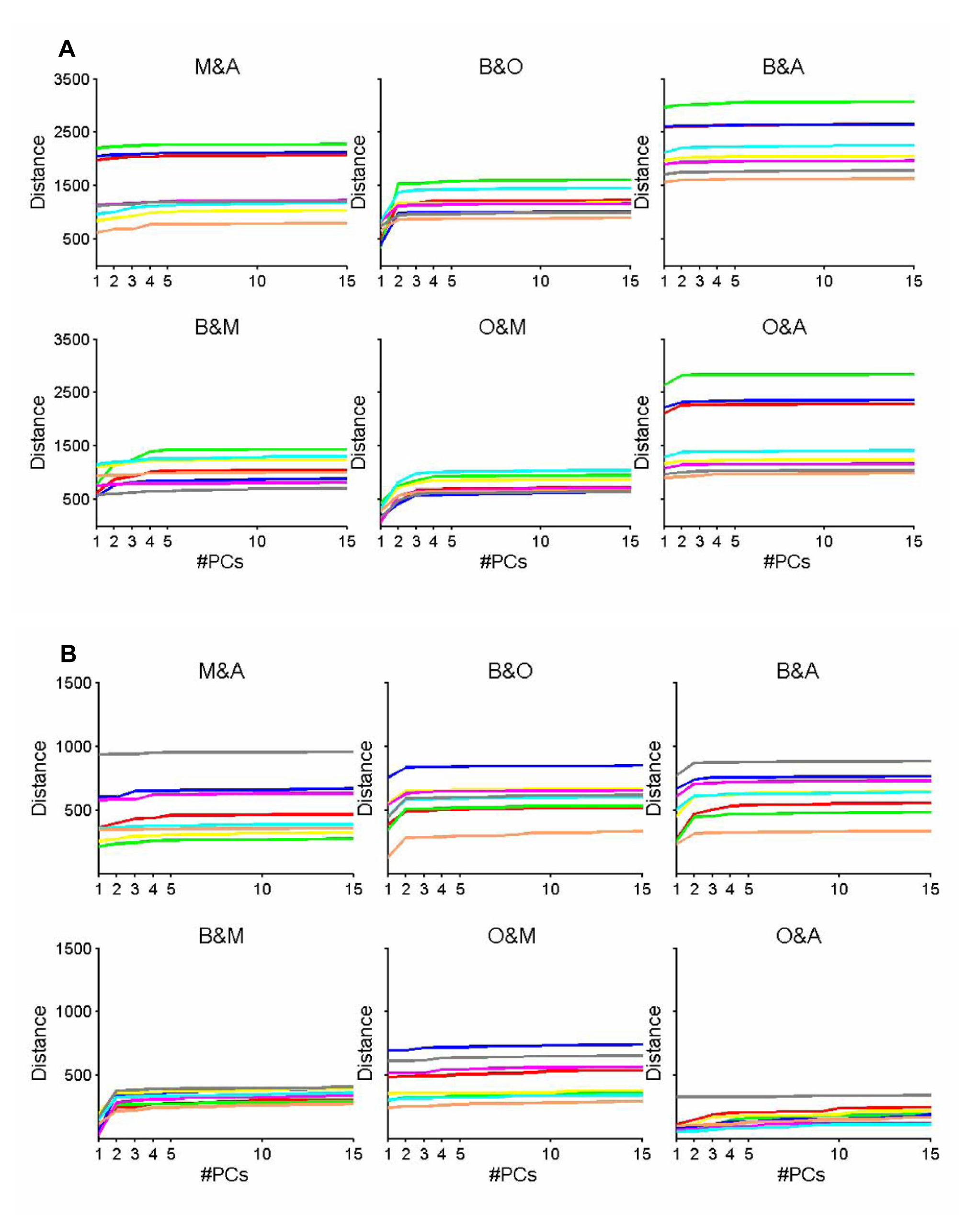

Supplement: Figure S3 — Validation of the three-PC based Euclidian distance measures. Euclidian distances of odour-evoked activity (A: sensory neurons, B: projection neurons) are computed for each pair of odours based on increasing numbers of principle components (x-axis: #PCs). The differently colored lines indicate data from individual animals. Note that for both populations of neurons the Euclidian distances remain constant or only slightly increase when using more than three principle components, demonstrating that the relative similarity between calcium activity patterns is effectively covered by the first three principle components. In other words, additional principle components do not add significant information. (TIF) [file pone.0024300.s003.tif]

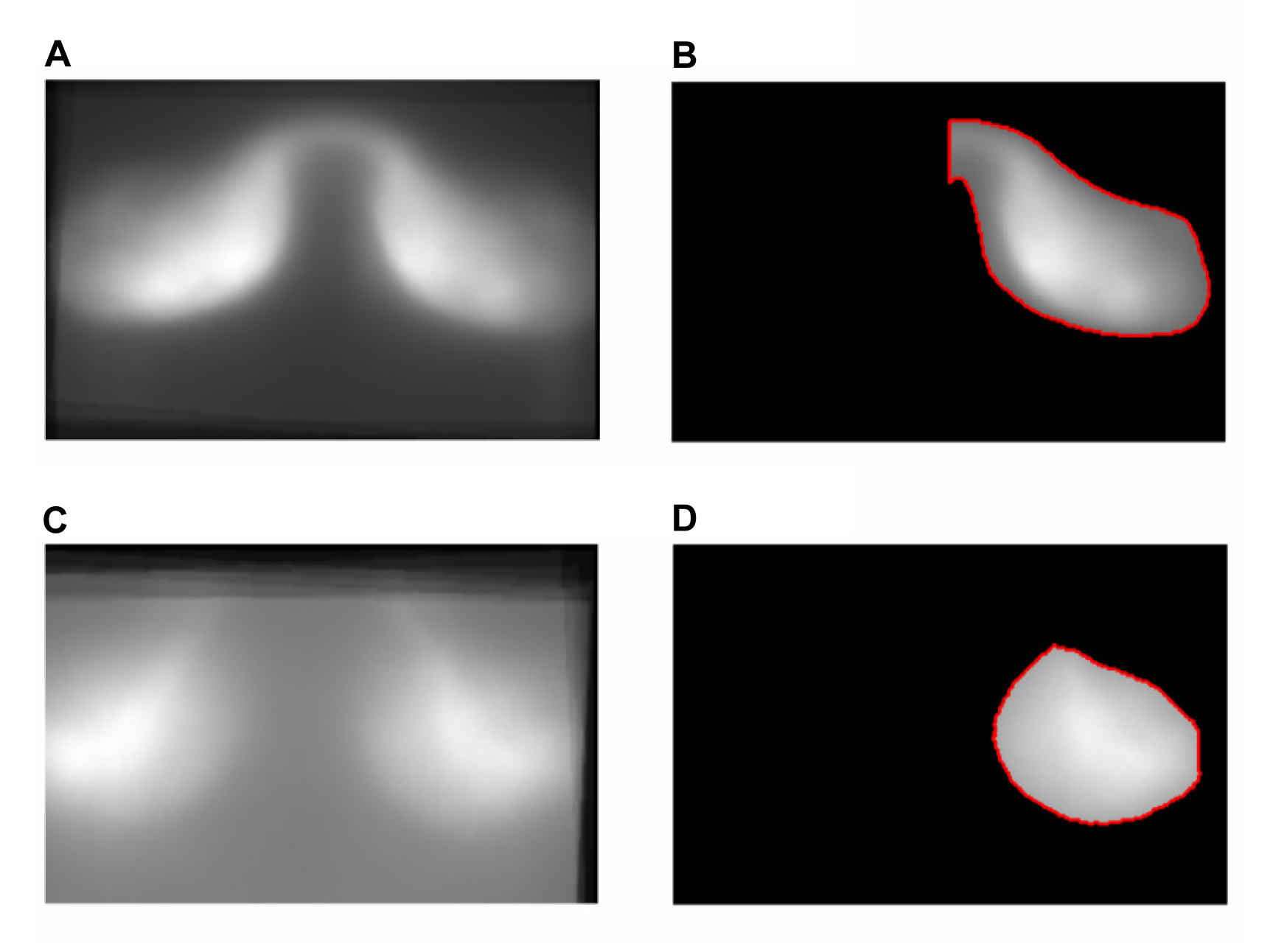

Supplement: Figure S4 — Definition of the Region of Interest (ROI) for the pixel-based PCA. (A) To define the Region of Interest (ROI) for the PCA of the sensory neurons innervating the antennal lobes across measurements, EYFP emission across 8 individual flies is averaged. (B) The region of interest used for PCA of sensory neuron activity in the antennal lobe (red circumference-line), defined by using a threshold of 0.45 of the maximum intensity value. (C) As in (A), but for the projection neurons. (D) As in (B), but for the projection neurons, except that (C, D) used a threshold of 0.60 of the maximum intensity value. (TIF) [file pone.0024300.s004.tif]

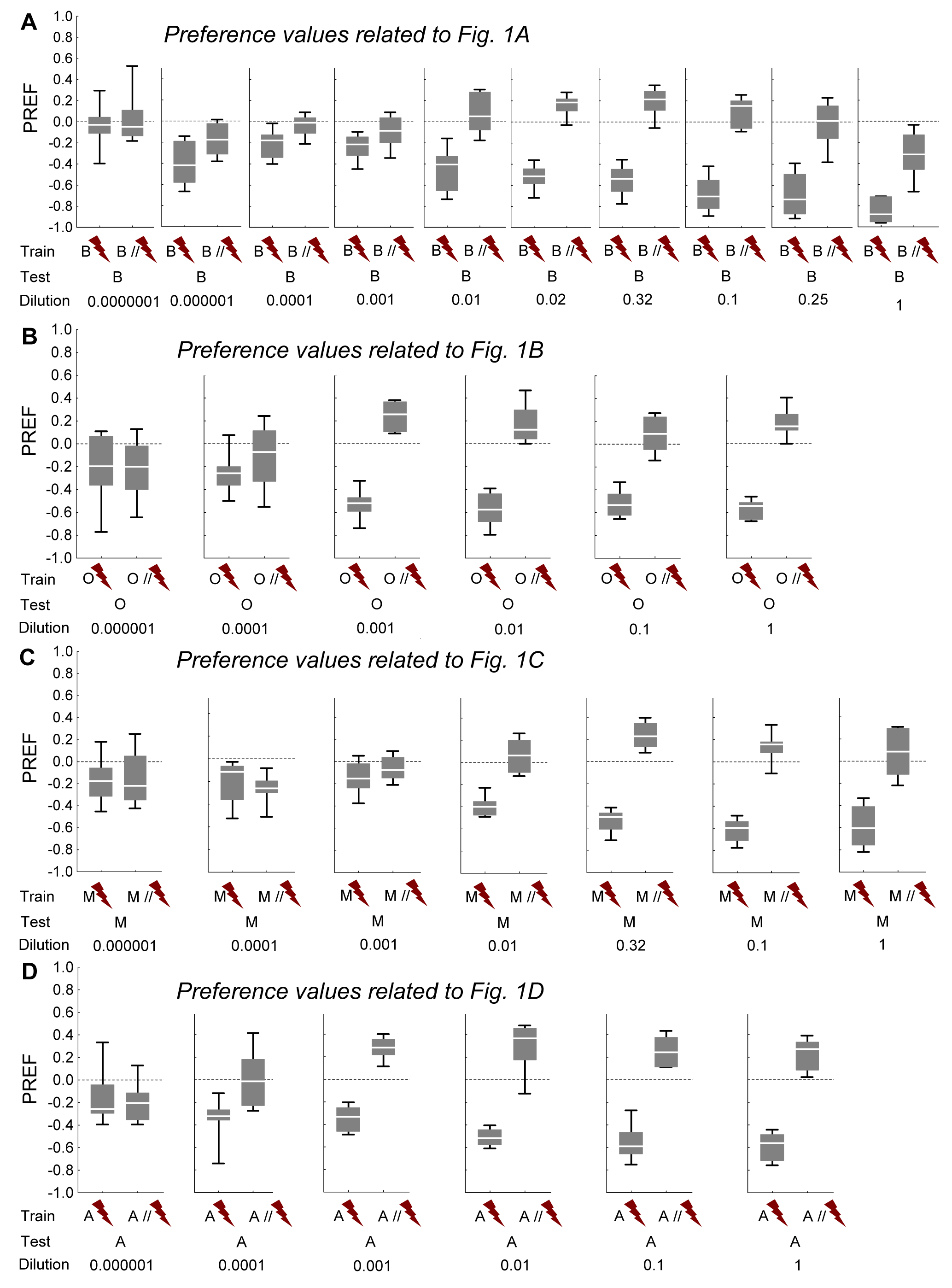

Supplement: Figure S5 — Preference scores underlying the associative performance indices shown in Figure 1A–D . The behaviour of the reciprocally trained groups of flies as underlying the associative learning indices (LIs) of Fig. 1A–D is documented by preferences (PREF) scores. On the basis of the the number of flies in the respective arm of the maze (#) these scores are calculated as: (TIF) [file pone.0024300.s005.tif]

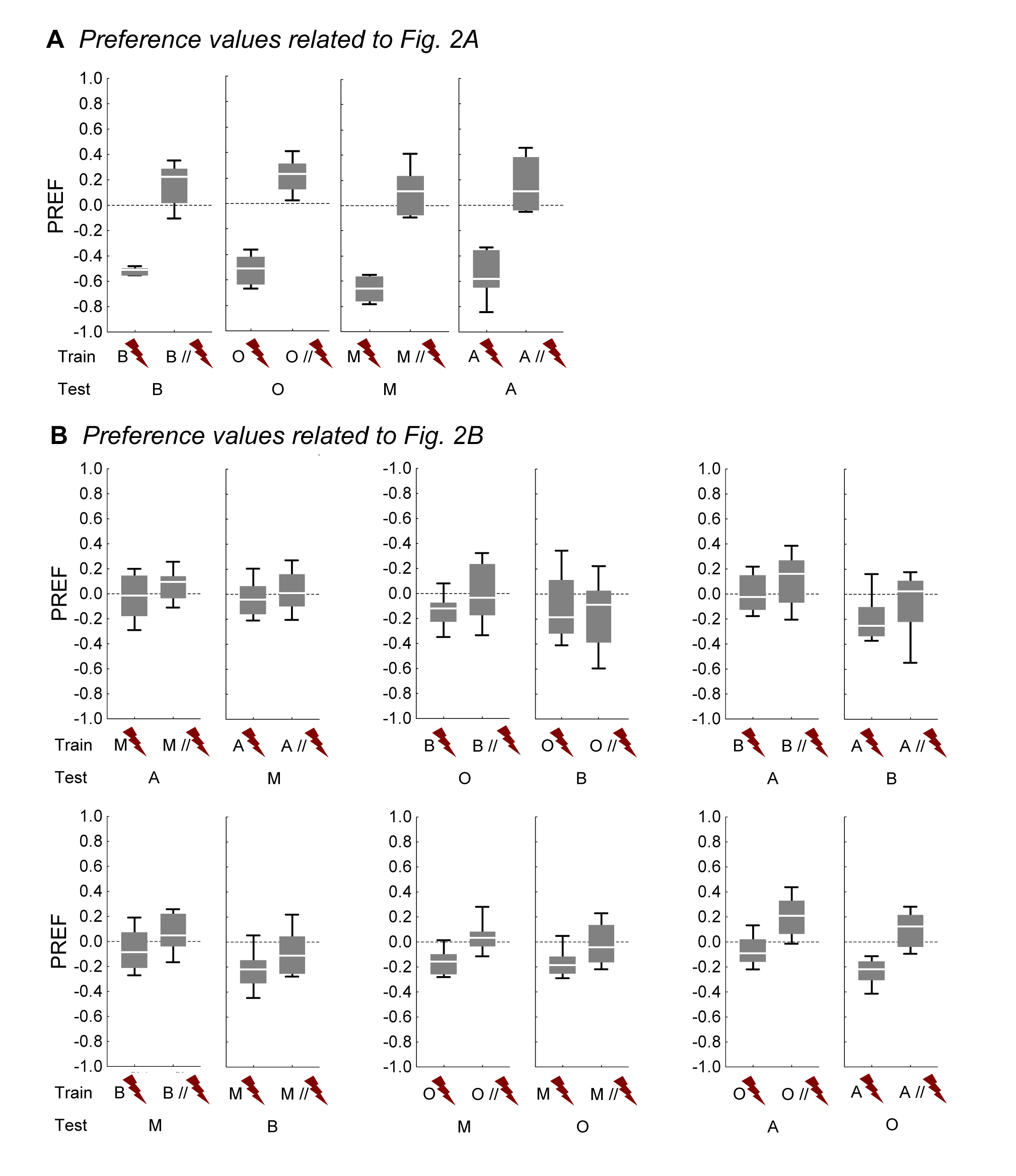

Supplement: Figure S6 — Preference scores underlying the associative performance indices shown in Figure 2A–B . The behaviour of the reciprocally trained groups of flies as underlying the associative learning indices (LIs) of Fig. 2A–B is documented by preferences (PREF) scores. On the basis of the the number of flies in the respective arm of the maze (#) these scores are calculated as: (TIF) [file pone.0024300.s006.tif]

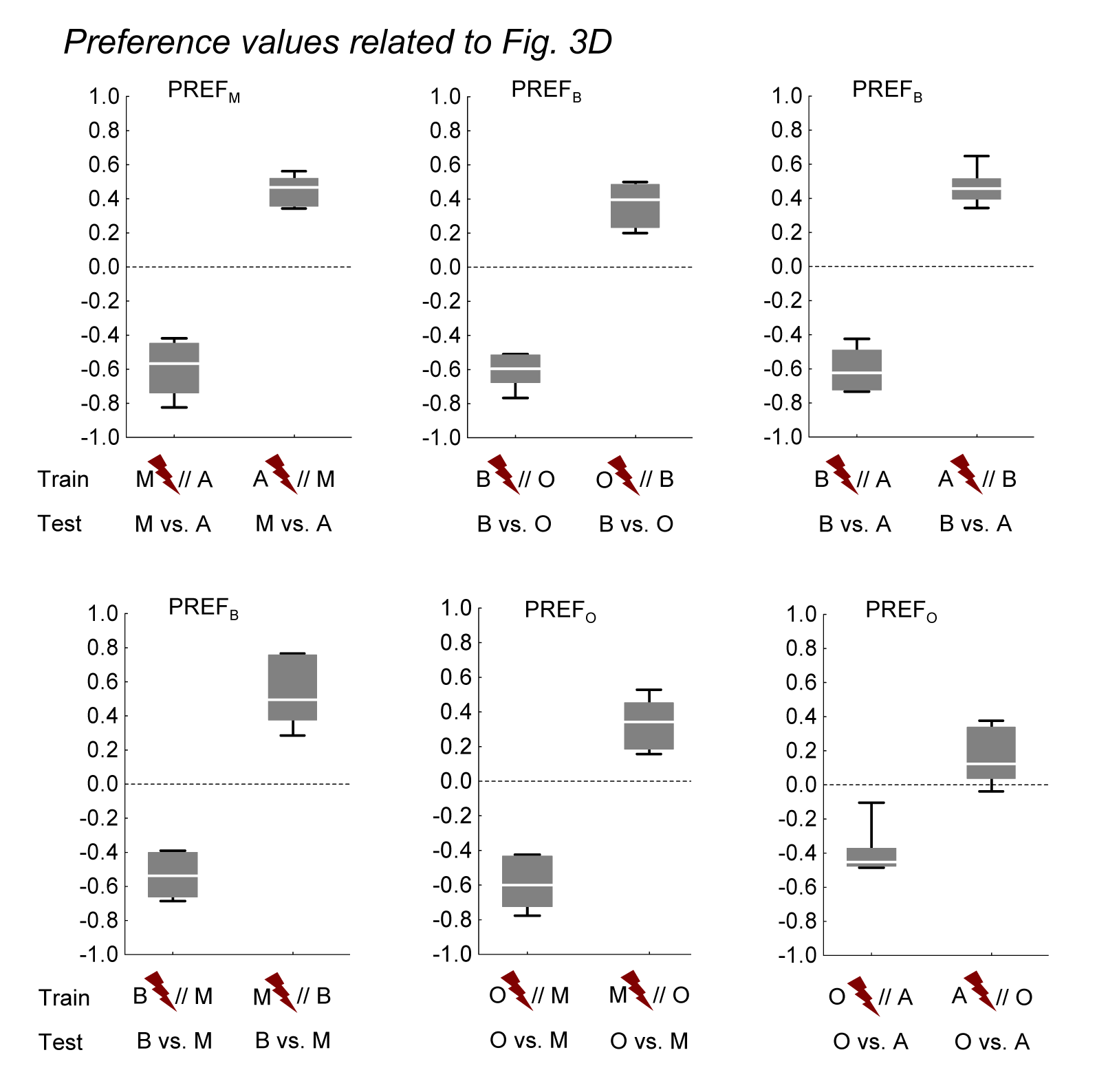

Supplement: Figure S7 — Preference scores underlying the associative performance indices shown in Figure 3D . The behaviour of the reciprocally trained groups of flies as underlying the associative learning indices (LIs) of Fig. 3D is documented by preferences (PREF) scores. On the basis of the the number of flies in the respective arm of the maze (#) these scores are calculated as: (TIF) [file pone.0024300.s007.tif]

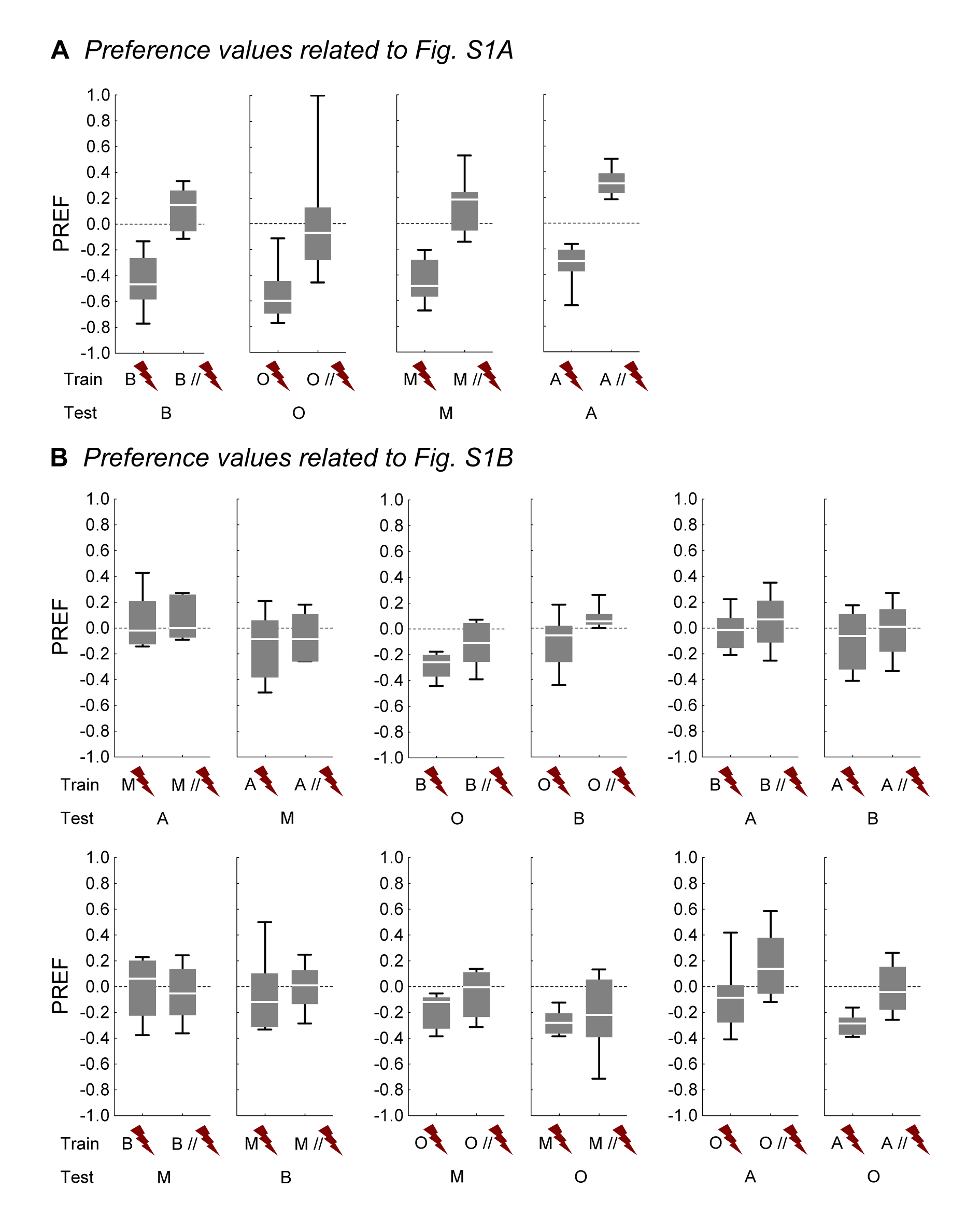

Supplement: Figure S8 — Preference scores underlying the associative performance indices shown in Figure S1A–B. The behaviour of the reciprocally trained groups of flies as underlying the associative learning indices (LIs) of Fig. S1A–B is documented by preferences (PREF) scores. On the basis of the the number of flies in the respective arm of the maze (#) these scores are calculated as: (TIF) [file pone.0024300.s008.tif]

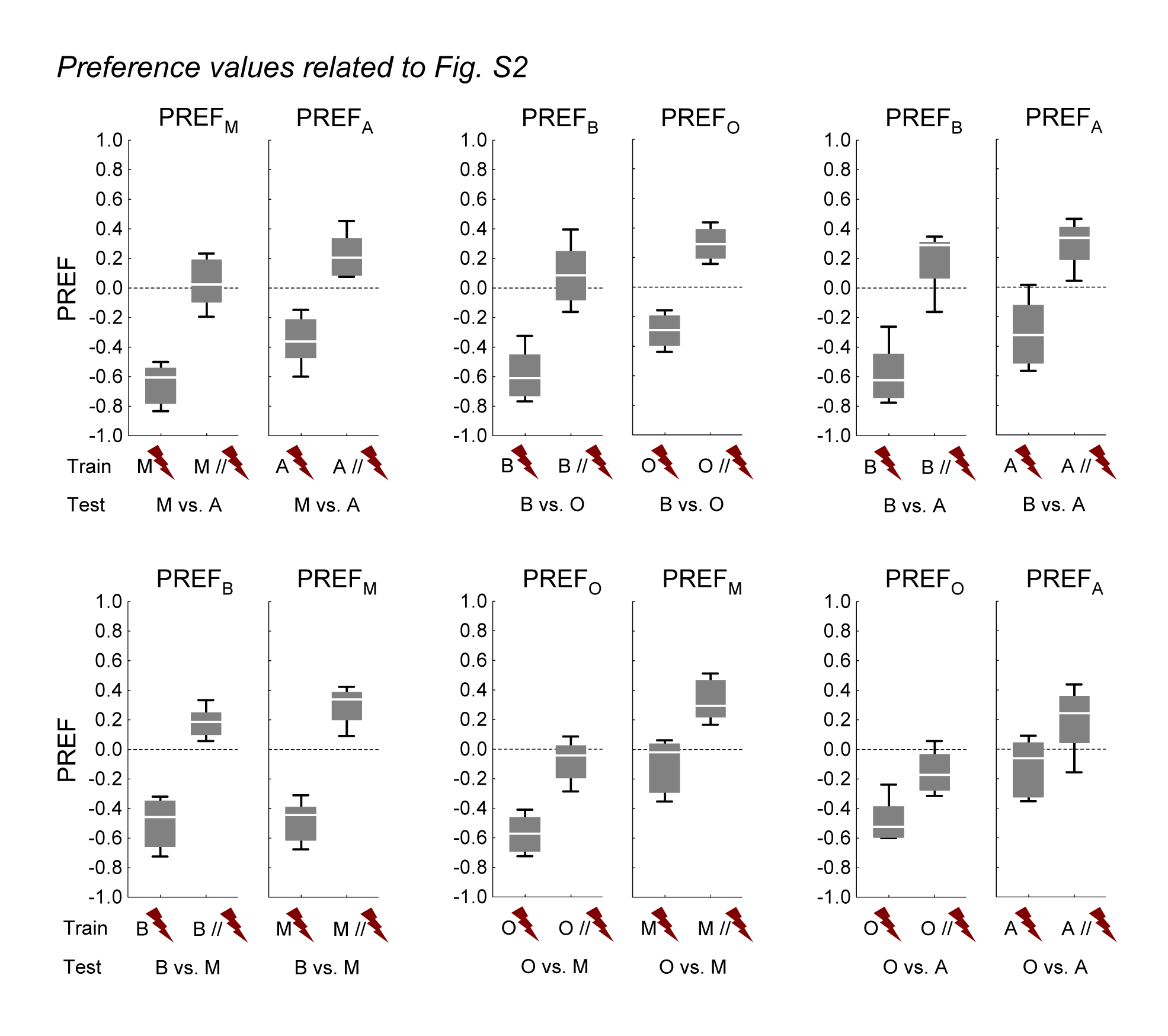

Supplement: Figure S9 — Preference scores underlying the associative performance indices shown in Figure S2. The behaviour of the reciprocally trained groups of flies as underlying the associative learning indices (LIs) of Fig. S2 is documented by preferences (PREF) scores. On the basis of the the number of flies in the respective arm of the maze (#) these scores are calculated as: (TIF) [file pone.0024300.s009.tif]
